# A Case Report on Endometrial Receptivity Array Test for Infertile Patient to Enhance Reproductive Outcomes

**DOI:** 10.7759/cureus.55059

**Published:** 2024-02-27

**Authors:** Muskan Khan, Akash More, Pranita A Bawaskar, Avanti Kalbande, Nancy Nair

**Affiliations:** 1 Clinical Embryology, School of Allied Health Sciences, Datta Meghe Institute of Higher Education and Research, Wardha, IND; 2 Clinical Embryology, Datta Meghe Medical College, Datta Meghe Institute of Higher Education and Research, Wardha, IND; 3 Obstetrics and Gynaecology, Datta Meghe Medical College, Datta Meghe Institute of Higher Education and Research, Wardha, IND

**Keywords:** window of implantation, recurrent implantation failures, endometrial receptivity array, endometrial receptivity, infertility

## Abstract

The issue of infertility affects couples all over the world. Recurrent implantation failure (RIF) is caused by immunology, thrombophilias, endometrial receptivity, microbiota, anatomical anomalies, male factors, and embryo aneuploidy. An accurate evaluation of endometrial receptivity (ER) in cases of RIF during in-vitro fertilization (IVF) treatments is crucial to improve reproductive outcomes. To find her accurate window of implantation (WOI), a 34-year-old woman with unexplained RIF underwent an endometrial receptivity array (ERA) test. This case study examines her inexplicable RIF and reproductive results. The ERA test examined gene expression patterns in endometrial tissue to determine the receptive phase for proper embryo transfer. Primary infertility, ineffective intrauterine insemination (IUI), and several unsuccessful IVF rounds were all part of the patient's medical history. Her WOI determined the embryo transfer timing after getting the ERA test results. The patient's clinical pregnancy was successful. This particular case focuses on the potential of the ERA test to improve reproductive outcomes. However, when using this strategy, it is essential to consider difficulties, including invasiveness and related expenses. In this case, the positive results urge future research to apply customized WOI determination using the ERA test to improve the effectiveness of IVF therapies in patients with recurrent implantation failure. More extensive investigations and controlled trials are required to confirm these results and the broader applicability of this strategy. The ERA test is promising, but to provide a holistic approach to infertility care, it should be taken into account together with endometrial changes and elements of embryo-endometrial interaction that impact the success of implantation.

## Introduction

Infertility is elucidated as the incompetence of a couple to culminate their genes into offspring despite repetitive one year of unprotected sexual encounters [1]. The two types of infertility are primary and secondary infertility. Primary fertility is a woman's inability to become pregnant through regular sexual activity [2]. Secondary infertility occurs when at least one prior pregnancy has been achieved. Embryo implantation is a complex and taxing process [3]. Effective implantation requires a good-quality embryo and a receptive endometrium. Due to the lack of reliable and unbiased testing methods, ER is seldom investigated in infertile patients or before In vitro fertilization (IVF) [4].

The receptivity of the endometrium is a sign that the endometrium has reached a stage of development where it is prepared to receive the well-formed embryo. It corresponds to WOI, which happens during the endometrial secretory phase and lasts three to six days (±28 days) of the menstrual cycle [5]. The WOI can be shortened or altered during the menstrual cycle under certain circumstances, such as inflammation or uterine anatomical abnormalities, which would preclude optimal implantation. RIF and infertility are common side effects of uterine disorders [6].

Recurrent implantation failure is a condition that causes infertility despite the transfer of more than three high-quality embryos in three or more IVF rounds [7]. Endometrial receptivity differs from patient to patient according to advanced maternal age, RIF, chronic inflammatory diseases, and hormonal imbalance, which was held to be the same in all patients. Endometrial receptivity peaks during the implantation window, lasting between 4-5 days (±28 days) of the menstrual cycle [8]. This condition has been attributed to several factors, some of which may be functional, such as chronic endometritis, while others may be related to the uterus. These factors include the uterine septate, submucosa fibroids, the uterine niche after cesarean section, and intrauterine adhesions. Endometrial tissue is subjected to gene expression analysis to determine the window of implantation using an endometrial ERA [9].

Genetic testing of endometrial tissue can now be used to determine gene expression in each phase of the endometrium since it has been established that each step of the endometrium has gene expression. During the menstrual phase, gene expression shifts to down-regulate matrix metalloproteinases (MMPs) responsible for endometrial tissue shedding while up-regulating genes related to cell proliferation, extracellular matrix remodeling, and angiogenesis. Estrogen receptor expression increases in response to increasing estrogen levels, preparing the endometrium for subsequent phases [10]. The mid-proliferative phase sees further proliferation of endometrial cells with up-regulation of genes related to cell cycle progression, DNA replication, and mitosis. As the proliferative phase progresses, genes involved in glycogen synthesis, lipid metabolism, and mucin production increase, which supports embryo implantation. During the secretory phase, genes for glycogen accumulation, mucin secretion, and uterine gland development are up-regulated, with the mid-secretory phase focusing on genes associated with embryo receptivity, such as leukemia inhibitory factor (LIF), integrins, and cytokines. The late secretory phase represents peak endometrial receptivity, characterized by maximal expression of genes essential for embryo implantation. These expressions affect cellular and metabolic activity, blood coagulation, humoral immunity, and mitotic and mitotic activity in the endometrial tissue, determining the receptivity phase. The seven endometrial phases are menstrual, mid-proliferative, early-proliferative, early-secretory, late-proliferative, mid-secretory, and late-secretory [11]. Depending on the phase, gene expression in these phases may change from down-regulation to up-regulation of the genes. These activities serve to define the window of implantation, and it is clear that the pre-receptive endometrium is related to the pre-receptive endometrium, which corresponds to the early secretory phase of the menstrual cycle immediately after ovulation, the receptive endometrium corresponds to the mid-secretory phase of the menstrual cycle, approximately 6-10 days after ovulation, and the post-receptive endometrium corresponds to the late secretory phase of menstrual cycle, approximately 10-14 days of menstrual cycle [12].

## Case presentation

Patient-related particulars

This case study focuses on a couple who, in June 2021, decided to go to the Wardha test tube baby facility in Sawangi, India, to meet their infertility-related requirements. The female patient, who was 34 years old, had been married for six years and had experienced primary infertility for five years. The patient was a homemaker, and the 40-year-old husband was a businessman. Both partners did not have habits of smoking, drinking alcohol, or using tobacco, and they did not use any other drugs.

Couple history

This study focuses on a nulligravida woman; they were married for six years without experiencing sexual problems. She had infertility for five years. The female spouse had a regular menstrual cycle. The results of the semen examination showed that overall motility was 70%, above the lower reference limit outlined in WHO 2021 standards, with a count of 17 million cells per milliliter. The reports state that 8% of the sperm have a normal morphology (Table 1) [13]. The patient was diagnosed with infertility in 2016. He had never had a genetic anomaly or any of his family.

**Table 1:** Semen analysis pH: Potential of Hydrogen; ml: milliliter

Parameter	Findings	Reference value
Volume	2.5	1.4-5 ml
pH	7.4	7.2-7.8
Liquefaction	20 minutes	<30 minutes
Sperm count	17	>16 million/ml
Total Motility	70	42 (40-43)%
Progressive Motility	55	30 (29-31)%
Morphology	8	≥ 4%

The doctor evaluated that the couple also had a history of a timing method three times (the timing method is a therapy that determines the day of ovulation and modifies the timing of sexual activity) and IUI five times for the patient with undiagnosed infertility, but neither method resulted in pregnancy. Therefore, the doctor switched to in vitro fertilization. After doing four cycles of IVF attempts, all were unsuccessful. All timing methods, IUI, and IVF attempts failed before their treatment at Wardha Test Tube Baby Centre (WTTBC). At WTTBC, the patient was initially diagnosed with unexplained infertility. The patient enrolled in our facility in 2021 to receive additional evaluation and treatment.

Clinical findings

The patient underwent hormone tests and hysterosalpingography. In hormone testing- the patient had an anti-mullerian hormone (AMH) value of 4.67 ng/mL, a sign of a healthy ovarian reserve [14]. It was common to have an antral follicle count (AFC). The progesterone value was 0.5 ng/mL, estrogen 98.435 pg/ml, the luteinizing hormone (LH) value was 2.56 mlU/ml, and the follicular-stimulating hormone (FSH) value was 5.62 mlU/ml. The thyroid stimulating hormone (TSH) is 4.8 mlU/L on hysterosalpingography, they demonstrated that there are no obstructions. However, no anomalies were seen. The results of the female hormonal profile are shown in Table 2.

**Table 2:** Blood hormone test of female patient AMH: Anti-Mullerian hormone; LH: Luteinising hormone; FSH: Follicular-stimulating hormone; TSH: Thyroid-stimulating hormone

Parameters	Reference values	Value
AMH	2 – 6.8ng/ml	4.67ng/ml
Progesterone	0.27 – 9.50ng/ml	0.5ng/ml
LH	0.8 – 7.6Miu/ml	2.56mIU/ml
FSH	1.5 - 12.4mIU/ml	5.62mIU/ml
TSH	0.4 – 4.0Miu/L	4.8mIU/l

Diagnostic assessment

We identified that the patient had RIF based on these clinical processes. Furthermore, after performing coagulation tests, antiphospholipid antibody tests, thyroid function blood tests, and hysteroscopy as part of the investigation of the RIF cause, no abnormalities were found in the patient. As a result, with the patient's informed consent, we attempted an ERA test to determine the WOI and change the date of embryo transfer.

Endometrial samples were taken from the uterine cavity using Pipelle catheters by a gynecologist for the ERA test on day P+5 of a hormone replacement treatment (HRT) cycle. After five complete days of progesterone impregnation, the endometrial biopsy (EB) is performed on the sixth day of the HRT cycle. After the biopsy, the endometrial tissue was placed in a cryotube with 1.5 ml of an RNA stabilizing agent, forcefully shaken for a short period, and refrigerated at 4°C for 4 hours, provided fluid was needed to cover the tissue completely. RNA breaks down when there is too much tissue present, while sufficient RNA is not available for extraction when there is insufficient tissue. The samples were then transported to a test facility in India at room temperature. Within two weeks, the test results were available. The response or non-receptivity of the endometrium was assessed using the ERA test. Figure 2 shows the result of the ERA test: The personalised embryo transfer (pET) of a blastocyst/s should be performed with 135 ±3 hours of progesterone administration (one day later than the time at which this endometrial biopsy was performed).

**Figure 1:**
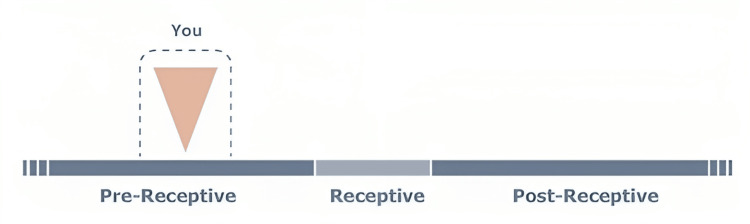
Pre-receptive ER test report of female patient ER: Endometrium receptivity

**Figure 2:**
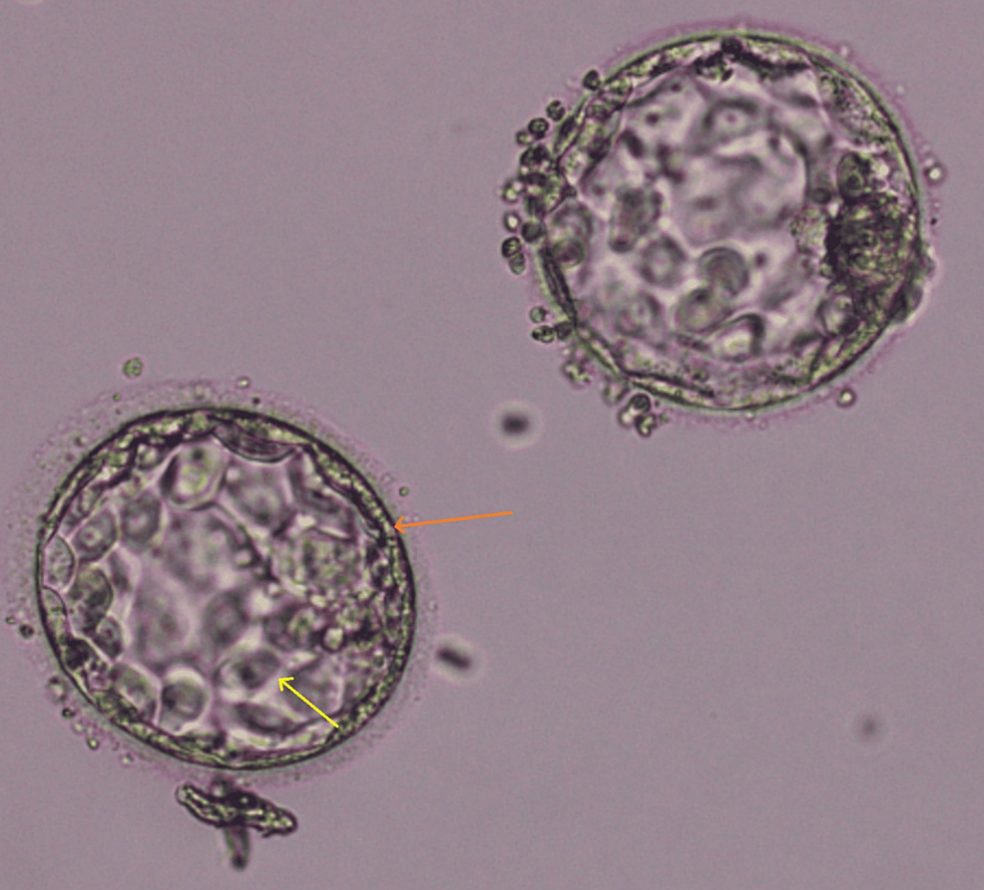
Day 5 Blastocyst of grade 3AB selected for ET 3: size of blastocyst; A: Trophectoderm (orange arrow); B: inner cellular mass (yellow arrow); ET: Embryo transfer

The patient's first cycle of IVF treatment included a gonadotrophin-releasing hormone (GnRH). Subsequently, we gave the patient a subcutaneous injection of 10.000 IU of human chorionic gonadotrophin (hCG). After 36 hours after injection delivery, we attempted transvaginal ovum pick-up. However, throughout the procedure, 12 oocytes were recovered, of which two were oocytes found to be GV, three were M1 phase, and seven were M2 phase oocytes. Frozen embryo transfer (FET) during a later HRT cycle triggers the ERA cycle. FET was performed in patients with a different WOI, using the customized WOI discovered by ERA (pET). Only two good-quality blastocysts of grade 3AB (Figure 2) were chosen for transfer out of the five retrieved blastocysts.

Follow-up therapeutical applications and diagnostic evaluation

Following successful embryo implantation, the patient was discharged with instructions to relax and avoid intense exercise and heavy lifting. In addition, calcirol sachets to increase her calcium intake, prednisolone (5 mg), progesterone 400 mg daily once, estradiol 2 mg tablet twice a day, vitamin E supplement once a day, multivitamins supplements, and iron supplements, told to take 500 mg of hydroxyprogesterone, intralipid injection, and beta-human chorionic gonadotrophin (β-hCG) injectables. The patient's blood sample was collected for a β-hCG test two weeks after embryo transfer (ET). A 322 mIU/ml concentration of β-hCG was measured, showing a positive pregnancy. Four weeks later, the patient was advised to come for a follow-up ultrasound, and the fetus showed a normal growth rate. The patient was instructed to continue taking her prescribed medications.

Timeline

The patient and her partner began trying to conceive naturally in 2015. After facing a year and a half of infertility, they consulted a doctor. The patient underwent five cycles of IUI using ovulation-stimulating medications from "April 2017 to December 2019". However, none of the IUI procedures leads to conception. The doctor suggested IVF treatment. The patient had her first cycle of IVF. Only 12 oocytes were extracted. Of 12 oocytes, only seven were fertilized, grown into embryos, and cryopreserved. A single embryo was transferred to end the cycle. However, the pregnancy test was negative. Again, from "March 2020 to February 2021", the patient underwent her second, third, and fourth IVF cycles, where all good-quality embryos were transferred, which were graded based on Gardner's grading system. However, all embryos failed to implant, resulting in negative pregnancy tests. A specialist considered examining her uterine receptivity with an ERA test in June 2021. The patient had her fifth IVF cycle using the customized embryo transfer time advised by the ERA test. Based on the timing suggested, two embryos were transplanted in "September 2021". Fourteen days later, β-hCG tested positive.

## Discussion

In this case, the patient was a 34-year-old female with primary infertility, a cause of unexplained recurrent implantation failure. The patient was advised to undergo an ERA test. Genetic analysis is performed on the findings of the ERA test. The patient's WOI period can be determined, and the ideal time for embryo transfer can be adjusted if we discover that the ERA test has moved the patient's WOI. As a result, an improvement in the reproductive results would be anticipated, as embryo transfer may be carried out at the proper time by defining the WOI period at the genetic level. As a result of the preceding, the ERA test led to the development of precision-based reproductive medicine.

The patient also developed a clinical pregnancy. These optimistic findings imply that the ERA test may have helped this patient with unexplained recurrent implantation failure undergo successful IVF [15]. The ERA test may have aided in successful implantation and subsequent clinical pregnancy by determining the WOI. The findings of this case report are consistent with other studies on applying the ERA test to improve IVF outcomes in patients with recurrent implantation failure [14,16]. Ahmed Bahgat et al. explained that patients with repeated unexplained implantation failure may benefit from tailored embryo transfer following endometrial receptivity array testing to determine their window of implantations [8].

Maria Ruiz-Alonso et al. concluded in this study that ER can only be accurately determined using the ERA test. Since it can be repeated and does not change over an extended period (1-2 years), it does not need to be redone in the case of a delay in treatment. Women who have experienced even one IVF failure after transferring two high-quality embryos should rule out a modified WOI. Establishing a responsive window will prevent embryo waste and other problems with financial distress, emotional and physical [11].

Hashimoto et al. concluded in this study that there is relevance in trying to identify their window of implantation (WOI) using the ERA in patients with unexplained RIF, considering the percentage of those who were not receptive (NR) and the pregnancy rates that resulted from the pET. More pregnancy rates are expected when euploid embryos are transferred in a personal WOI [17]. Rubin et al. say that ERA is a method for determining endometrial receptivity. Endometrial biopsies are required for ERA and may be paired with additional endometrial biopsies for pathological analysis. The ERA uses molecular arrays to "date" the endometrium and assess endometrial growth and differentiation. As a result, the ERA can help define a tailored WOI, which can then be used in a patient's FET plan to maximize the likelihood of a successful transfer [18].

Scott Jr et al. explained that the transfer of healthy, high-grade embryos did not ensure a healthy pregnancy; as a result, the term unexplained recurrent implantation failure was coined. Transferring healthy high-grade embryos during the implantation window can help reduce the failure rate by some amount [19]. Hashimoto et al. in conclusion, in this study, by analyzing gene expression in endometrial tissue to evaluate receptivity, the problem of identifying the window of implantation has been solved. According to numerous studies on the endometrial receptivity array, the WOI had been displaced in about 25% of cases of implantation failure. This implied the need to start pET with the diagnosed WOI according to the ERA test to discover the importance of clinical application [17].

The ERA test has a specific limitation, focusing primarily on using gene expression patterns to assess the uterine receptivity window. However, it might not consider additional variables that can affect the success of implantation, such as the local uterine environment, immunological factors, or other molecular interactions. Variations in the sampling procedure and the dynamic nature of the endometrial environment may also impact the test's reliability. And the cost, the requirement for embryo vitrification, and the degree of invasiveness of the procedure [20]. It should be highlighted that this case study only looks at one subject. More studies, including larger patient populations and controlled trials, must verify these findings.

## Conclusions

ERA is one of the tests for ER that may accurately and objectively diagnose ER. It has been utilized to build a customized WOI for each patient by defining a changed WOI. It has shown advantages in enhancing reproductive function in patients with RIF. However, more studies are required to confirm these early results. Its intrusiveness and associated costs limit it.
